# Meta-Analysis of Interactions between Arbuscular Mycorrhizal Fungi and Biotic Stressors of Plants

**DOI:** 10.1155/2014/746506

**Published:** 2014-01-16

**Authors:** Haishui Yang, Yajun Dai, Xiaohua Wang, Qian Zhang, Liqun Zhu, Xinmin Bian

**Affiliations:** ^1^College of Agriculture, Nanjing Agricultural University, No. 6 Tongwei Road, Jiangsu Province, Nanjing 210095, China; ^2^Research Institute of Forestry, Chinese Academy of Forestry, Beijing 100091, China

## Abstract

Naturally, simultaneous interactions occurred among plants, herbivores, and soil biota, that is, arbuscular mycorrhizal fungi (AMF), nematodes, and fungal pathogens. These multiple interactions play fundamental roles in driving process, structure, and functioning of ecosystems. In this study, we conducted a meta-analysis with 144 papers to investigate the interactions between AMF and plant biotic stressors and their effects on plant growth performance. We found that AMF enhanced plant tolerance to herbivores, nematodes, and fungal pathogens. We also found reciprocal inhibition between AMF and nematodes as well as fungal pathogens, but unidirectional inhibition for AMF on herbivores. Negative effects of AMF on biotic stressors of plants depended on herbivore feeding sites and actioning modes of fungal pathogens. More performance was reduced in root-feeding than in shoot-feeding herbivores and in rotting- than in wilt-fungal pathogens. However, no difference was found for AMF negative effects between migratory and sedentary nematodes. In return, nematodes and fungal pathogens generated more reduction of root colonization in Non-Glomeraceae than in Glomeraceae. Our results suggested that AMF positive effects on plants might be indirectly mediated by competitive inhibition with biotic stressors of plants. These positive and negative interactions make potential contributions to maintaining ecosystem stability and functioning.

## 1. Introduction

In natural ecosystems, species interactions form a complex web of associations [1]. Traditionally, the aboveground component of ecosystems is considered in isolation from belowground [2]. However, there is now increasing recognition that both components interact closely with one another [3]. For example, plants interact simultaneously with aboveground insect herbivores and soil biota, such as arbuscular mycorrhizal fungi (AMF), nematodes, and fungal pathogens [4]. These interactions between aboveground and belowground play vital roles in controlling ecosystem properties and processes [3].

AMF are ecologically important components in soil communities [5] and form mutualistic interactions with roots of most land plants [6]. For this two-way interaction, host plants provide carbohydrates to AMF in return for several benefits of nutrients and signals [6]. AMF extensive hyphal networks could explore more soil volume where roots could not arrive [6]. Through hyphal network, by vividly described as “superhighways” [7], AMF could transport phosphorus, nitrogen, sulfurous, water, and microelements [6, 8–10]. Especially, one recent study reported that AMF mycelial network carried defensive signals to neighbor plants [11].

Herbivores negatively interact with plants [12]. Herbivores removed plant biomass and reduced photosynthetic area, while plants evolutionarily obtained defensive systems to herbivores [12]. Increasing studies suggested that AMF could modify the pairwise interactions between plants and herbivores [1]. In fact, the three-way interactions among AMF, plants, and herbivores occurred simultaneously in natural conditions [1]. Controversial results were reported for the direction of AMF-mediated plant-herbivore interactions. For example, positive effects were reported for water weevil-rice interactions [13] and weevil-clover interactions [14], negative effect for black vine weevil-strawberry interactions [15], and neutral effect for *Junonia coenia*-*Plantago lanceolata* interactions [16]. This high variation was possibly caused by feeding modes or dietary breadth of herbivores [17]. Some hypotheses have been used to explain AMF-mediated plant-herbivore interactions. For AMF positive effects on plant-herbivore interactions, it was hypothesized that AMF improved plant quality for herbivores [13, 14]; for AMF negative effects on plant-herbivore interactions, it was assumed that AMF induced plant defense responses and increased chemical resistance [4, 11], changed palatability of plants to herbivores [18], or interacted with fungal endophytes to inhibit herbivores [19]. In return, herbivores possibly suppress AMF by affecting carbon allocation of plants [20].

Another pairwise negative interactions occurred between plants and soil pathogens, such as nematodes and pathogenic fungi [5]. Nematodes and pathogenic fungi cause serious plant diseases, especially for crops [21–23]. However, there is now more recognition that AMF could modify this negative interaction by directly or indirectly interacting with these plant biotic stressors [1, 5]. AMF could directly compete for infection sites or host-derived carbon and thus suppress these pathogens [24–26]. However, most commonly, AMF indirectly interact with pathogens, such as enhancing plant tolerance by improving nutrition conditions or root growth [27, 28] or increasing resistance by inducing defensive responses [4, 29]. AMF could also alter plant root exudations used by pathogens [30], as well as priming other soil microbes which are antagonist of pathogens [31]. However, nematodes and fungal pathogens could also reduce AMF root colonization possibly through resource competition [5].

In this study, we conducted a meta-analysis with 144 papers to decipher the effects of interactions between AMF and biotic stressors of plants on plant performance. We hypothesized that AMF had negative effects on biotic stressors of plants and enhanced plant performance under these biotic stresses. Our questions are as follows: (1) whether AMF promote plant growth under attacks by herbivores, nematodes, and pathogenic fungi, (2) whether AMF have negative effects on these plant biotic stressors, and (3) whether these biotic stressors of plants affect AMF.

## 2. Methods

### 2.1. Data Collection

We searched for published and unpublished studies about interactions between AMF and biotic stressors of plants in ISI Web of Science database (http://apps.webofknowledge.com/), Google Scholar (http://scholar.google.com.hk/), and CNKI database (http://www.cnki.net/). We concentrated on three groups of biotic stressors of plants: insect herbivores, nematodes, and fungal pathogens. Thus, our searching term combinations are as follows: (abuscular AND mycorrhiza*) AND (pathogen OR nematode OR herbivore*).

All papers for our meta-analysis should meet the following criteria: (1) studies should include pairwise control and experimental treatments; (2) studies should report the definite identity information of AMF, biotic stressors, and host plants; (3) the same host plant or AMF in different papers was treated as independent studies [32]; (4) the most recent data was collected for the same host plant-AMF pairs in different years; (5) for the same host-AMF pairs in different sites, habitats, or experimental techniques, each study was treated as independent data record. Totally, 144 papers satisfied with our selection criteria (see Supplementary Material Appendix-1 available online at http://dx.doi.org/10.1155/2014/746506).

We selected plant performance, biotic stressor performance, and AMF growth as responsive variables. Plant performance was reflected by shoot biomass or total biomass inoculated with and without AMF under biotic stress. Stressor performance was reflected by growth or reproduction inoculated with and without AMF. Herein, we used larval mass, number of eggs, or survival to reflect herbivores; nematode performance was reflected by galls or eggs per gram of root, or population density per volume of soil. Pathogen performance was represented by frequency of plant tissue necrosis or vascular discoloration. AMF growth was represented by root colonization rate with or without biotic stress.

We retrieved values of means, standard deviation or error (SD or SE), and sample size (*N*) from control and experimental treatments of each study. For data presented in tables, we directly extracted it; for data presented in figures, we digitized it with GetData software (http://getdata-graph-digitizer.com/). When SE was reported, we transformed SD with SE ∗ sqrt (*N*). We assumed that unknown error bars stand for SE. For these studies without presenting SD or SE values, we estimated it with the methods used by Van Groenigen et al. [33]. We first calculated the coefficient of variation (CV) for each dataset and then estimated the missing SD with the reported mean value by multiplying the averaged CV and squared it.

According to host plant functional groups, we categorized our data into grass, forb, and tree. Plant response to AMF interacting with biotic stressors possibly depends on its family identity [32]; thus, we also classified our data through plant family. For the dataset of herbivore stress, we classified our data into Asteraceae, Fabaceae, Plantaginaceae, Poaceae, and Rosaceae; for the dataset of nematode stress, we classified our data into Asteraceae, Cucurbitaceae, Fabaceae, Malvaceae, Oleaceae, Passifloraceae, Poaceae, Rosaceae, Rutaceae, Solanaceae, and Vitaceae; for the dataset of fungal pathogen stress, we classified our data into Cucurbitaceae, Fabaceae, Lamiaceae, Malvaceae, Poaceae, Rosaceae, Rutaceae, and Solanaceae. According to feeding site of insect herbivores, we categorized herbivore dataset into shoot-feeding and root-feeding. According to feeding modes of nematodes, we classified nematode dataset into migratory endoparasitic and sedentary endoparasitic. According to acting modes of fungal pathogen, we classified pathogen dataset into rotting- and wilt-type. In addition, each dataset was also categorized into Glomeraceae and Non-Glomeraceae according to functional diversity among AMF families [34] (Supplementary Material Appendix-2).

### 2.2. Meta-Analysis

We selected log response ratio (LnRR) to calculate effect size of predictory variables for each dataset. LnRR is a standardized, unit-less metric and has powerful statistical properties [32]. This metric is widely used in meta-analysis studies. LnRR was calculated as follows [35]:

(1) $$\begin{array}{c} \text{LnRR}=\ln\left(\frac{{\bar{X}}^{E}}{{\bar{X}}^{C}}\right). \end{array}$$

Here, ${\bar{X}}^{E}$and ${\bar{X}}^{C}$ represent the mean values of experimental and control treatments, respectively.

The variance of LnRR was calculated from the following equation:

(2) $$\begin{array}{c} v_{\text{LnRR}}=\frac{{\left({\bar{S}}^{E}\right)}^{2}}{N^{E}{\left(\bar{X^{E}}\right)}^{2}}+\frac{{\left({\bar{S}}^{C}\right)}^{2}}{N^{C}{\left(\bar{X^{C}}\right)}^{2}}. \end{array}$$

Here, $\bar{S}$ represents standard deviation and $\bar{N}$ represents sample number.

All meta-analyses were conducted in MetaWin 2.0 [35]. Radom effect model was selected for these analyses, because the model assumption is easily satisfied by ecological data [35]. We calculated weighted mean effect size (LnRR^+^) for each predictory variable. 95% confidence interval (CI) was calculated with 9999 bootstrap replications. If 95% CI includes 0, it is indicated that difference is not significant, or else significant. Difference between groups was inferred from whether 95% CIs were overlapped with each other. If both 95% CIs were not overlapped, it is suggested that difference was significant between both groups.

In addition, one important assumption of meta-analysis is data without publication bias. However, positive or negative ecological data was potentially biased to publication, while neutral data was not easily accepted to be published. Thus, we examined whether our datasets had publication bias. Two methods were used for this analysis: Spearman correlation analysis and fail-safe number. We first conducted Spearman correlation analysis between effect size and sample number. If no correlation was found, it is indicated that no publication bias in the dataset, or else publication bias existed in the dataset. We further used fail-safe number to infer whether the publication bias affects our conclusions. If fail-safe number > 5*N* + 10, suggesting that publication bias will not affect the overall conclusions.

## 3. Results

**Figure 1:**
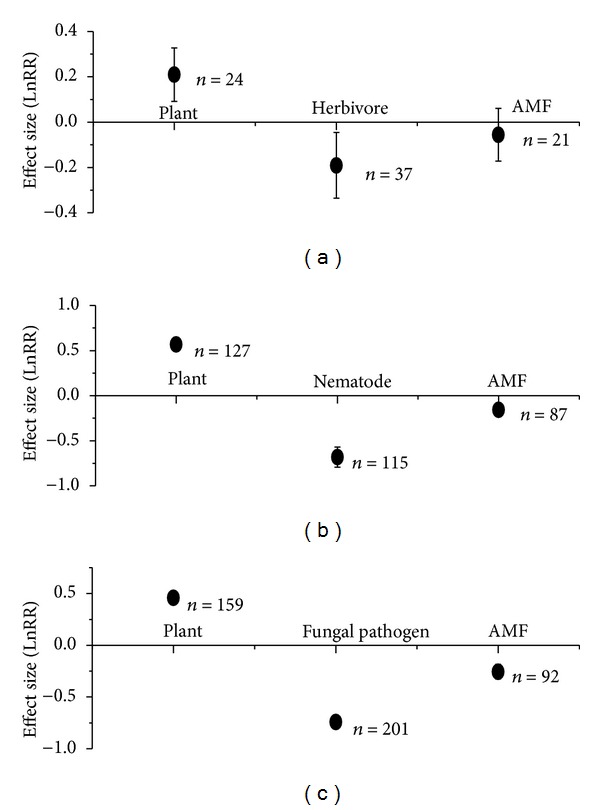
Effect size (mean ± 95%  CI) of plants, biotic stressors, and AMF. (a) Effect size of plant refers to plant growth response to AMF at herbivore attack; effect size of herbivore refers to herbivore performance response to AMF; effect size of AMF refers to AMF growth response to herbivores, (b) similar to (a) but under nematode attacks, (c) similar to (a) but at pathogenic fungi stress.

Totally, AMF significantly enhanced plant growth under attacks by herbivores, nematodes, and fungal pathogens (effect size with 95% CIs: 0.21 with 0.09~0.33; 0.57 with 0.49~0.64; 0.46 with 0.40~0.52, resp.) (Figure 1). For AMF-herbivore interactions, AMF significantly inhibited herbivore performance; however, herbivore did not significantly affect AMF growth (effect size with 95% CIs: −0.19 with −0.34~−0.05 for herbivore; −0.06 with −0.06~0.17 for AMF) (Figure 1(a)). For AMF-nematode interactions, both partners significantly inhibited each other (effect size with 95% CIs: −0.68 with −0.79~−0.60 for nematode; −0.16 with −0.22~−0.10) (Figure 1(b)). Similar pattern of reciprocal inhibition was found for AMF-fungal pathogen interactions (effect size with 95% CIs: −0.74 with −0.81~−0.68; −0.26 with −0.33~−0.18) (Figure 1(c)).

**Figure 2:**
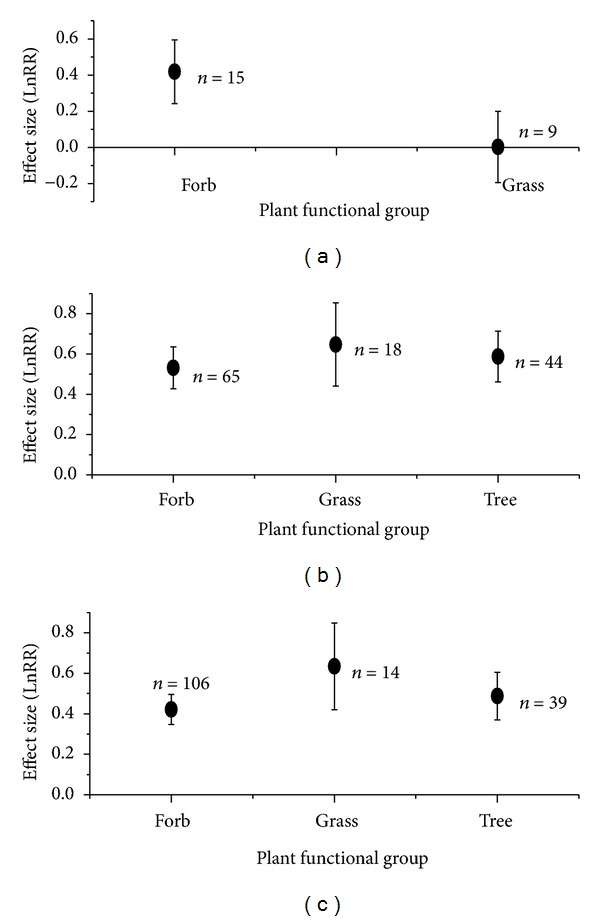
Effect size (mean ± 95%  CI) of plant functional groups to AMF under attacks of herbivores (a), nematodes (b), and fungal pathogens (c).

**Figure 3:**
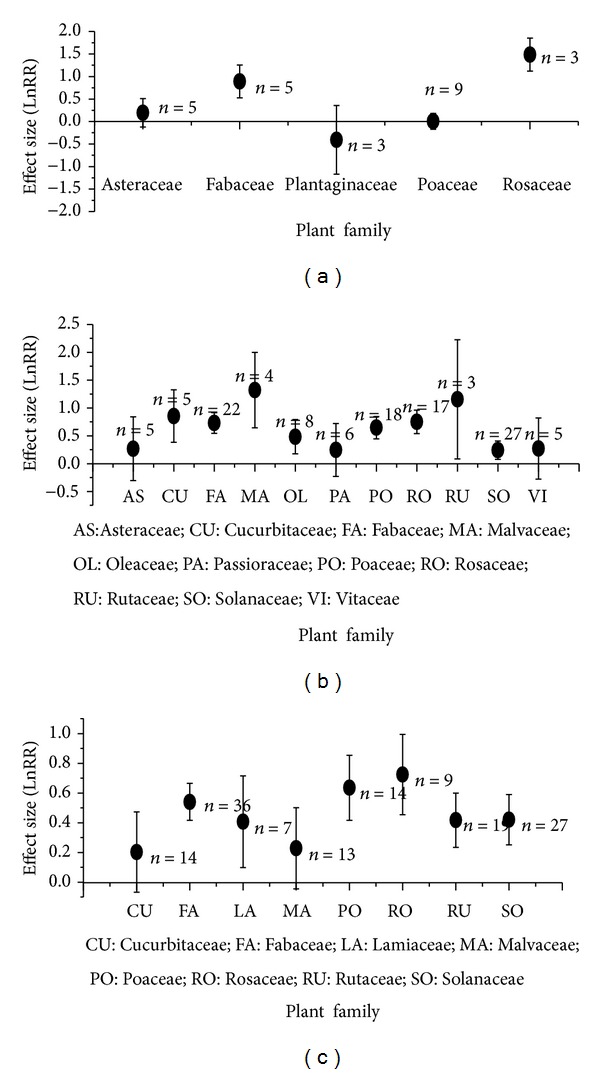
Effect size (mean ± 95%  CI) of plant family to AMF under attacks of herbivores (a), nematodes (b), and fungal pathogens (c).

Under biotic stress, AMF-mediated plant tolerance varied with its functional group and taxonomic family (Figures 2 and 3). At herbivore attack, AMF significantly enhanced growth performance of forbs, but it did not affect grass (effect size with 95% CIs: 0.42 with 0.24~0.60 for forb; 0.00 with −0.19~0.20) (Figure 2(a)). At nematode and fungal pathogen stress, AMF significantly increased growth performance of grass, forb, and tree, but no significant difference occurred among these functional groups (effect size with 95% CIs: 0.53 with 0.43~0.64, 0.65 with 0.44~0.85, and 0.59 with 0.46~0.71 for forb, grass, and tree under nematode stress; 0.42 with 0.35~0.50, 0.63 with 0.42~0.85, 0.49 with 0.37~0.61 for forb, grass and tree under fungal pathogen stress) (Figures 2(b) and 2(c)). In addition, plant family exhibited high variation to AMF under different biotic stress. At herbivore stress, AMF significantly enhanced growth response of Fabaceae and Rosaceae but not for other families (Figure 3(a)). At nematode stress, except for Asteraceae, Passifloraceae, and Vitaceae, AMF significantly enhanced growth of other nine plant families (Figure 3(b)). At fungal pathogen stress, except for Cucurbitaceae and Malvaceae, other six plant families exhibited significant growth response to AMF (Figure 3(c)).

**Figure 4:**
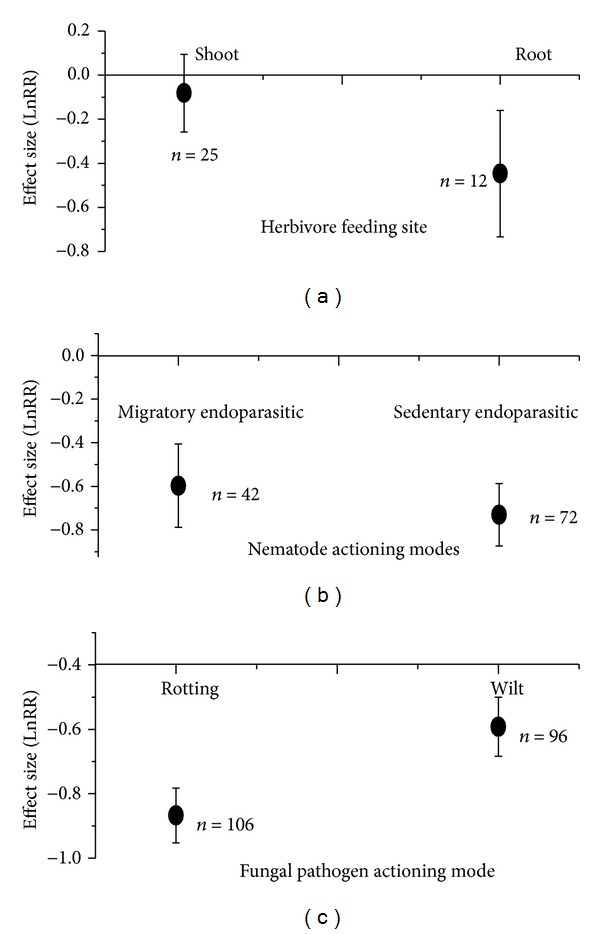
Effect size (mean ± 95%  CI) of biotic stressors of plants to AMF: (a) herbivores; (b) nematodes; (c) fungal pathogens.

**Figure 5:**
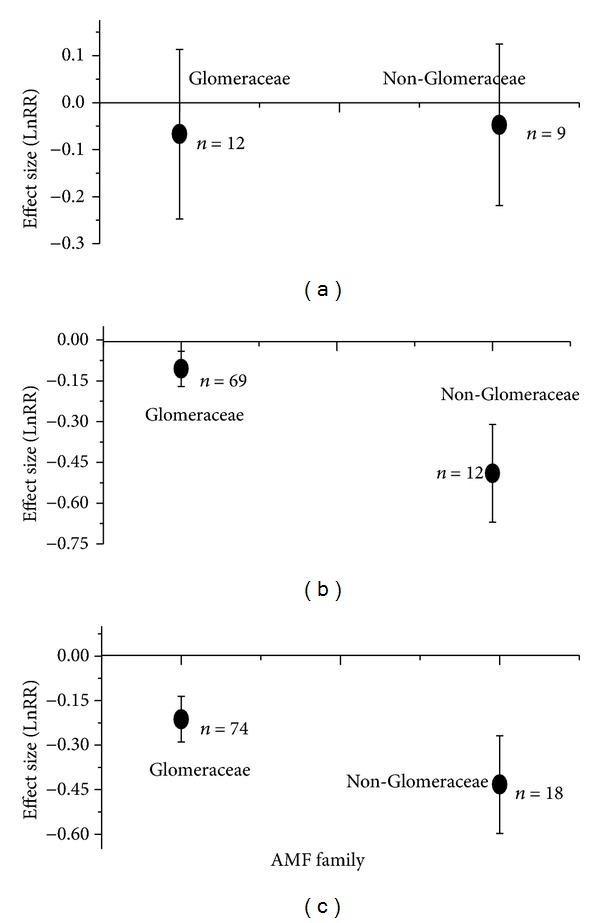
Effect size (mean ± 95%  CI) of AMF to biotic stressors of plants: (a) herbivores; (b) nematodes; (c) fungal pathogens.

AMF interact closely with herbivores, nematodes, and fungal pathogens (Figures 4 and 5). AMF significantly inhibited root-feeding herbivores but did not affect shoot-feeding ones (effect size with 95% CIs: −0.45 with −0.73~−0.16; −0.08 with −0.26~0.09, resp.) (Figure 4(a)). However, the effect of herbivores on AMF was not significant, irrespective of Glomeraceae or Non-Glomeraceae (effect size with 95% CI: −0.07 with −0.25~0.11; −0.05 with −0.22~0.13, resp.) (Figure 5(a)). AMF interacted with nematode and fungal pathogen with reciprocal inhibitions. AMF significantly reduced nematode growth performance, but this negative effect was not dependent on nematode feeding modes, that is, migratory endoparasitic or sedentary endoparasitic (effect size with 95% CIs: −0.60 with −0.79~−0.41; −0.73 with −0.87~−0.58, resp.) (Figure 4(b)). However, the negative effects of nematodes on AMF depended on fungal family. More reduction in Non-Glomeraceae occurred than in Glomeraceae (effect size with 95% CIs: −0.11 with −0.17~−0.04; −0.49 with −0.67~−0.31) (Figure 5(b)). For fungal pathogens, AMF caused more reduction in pathogen performance of rotting-type than in wilt-type (effect size with 95% CIs: −0.87 with −0.95~−0.78; −0.59 with −0.68~−0.50) (Figure 4(c)). Nonetheless, the negative effects of fungal pathogen depended on AMF family. Fungal pathogen had less inhibitive effects on Glomeraceae than on Non-Glomeraceae (effect size with 95% CIs: −0.21 with −0.29~−0.14; −0.43 with −0.60~−0.27) (Figure 5(c)).

## 4. Discussion

In managed and natural ecosystems, plants simultaneously interact with herbivores, AMF, nematodes, and fungal pathogens. These multiple interactions play important roles in maintaining the functioning and structure of ecosystems. In this meta-analysis, we found that AMF significantly enhanced plant growth irrespective of attacks by herbivores, nematodes, or fungal pathogens. Although simultaneously interacting with plants, reciprocal inhibition occurred between AMF and nematodes, as well as fungal pathogens. However, we found that AMF had negative effects on herbivores, while herbivores did not affect AMF growth.

This study found that AMF negative effects on herbivores depended on feeding sites. AMF significantly inhibited root-feeding herbivores, but did not affect shoot-feeding ones (Figure 4(a)). Our result is opposite to Currie et al. [14] as well as Koricheva et al. [17]. Currie et al. [14] found that AMF increased larval survival of root-feeding insects; Koricheva et al. [17] addressed that chewer insects benefited from mycorrhizae. However, Gange [15] provided strong support for our results that AMF significantly reduced the larval survival and biomass of root-feeding black vine weevil. These differences might be caused by diet breadth [17]. AMF possibly had more negative effects on generalist insect herbivores than on specialist ones [14, 17]. Negative effects of AMF on root-feeding herbivores might be mediated by inducing chemical defensive system [4], altering root morphology and physiology [5] or interacting with fungal endophytes [19]. In return, herbivores possibly suppress AMF by reducing photosynthate allocation with shoot or root removal [20]. Barto and Rilling [20] found that herbivores totally reduced AMF growth by 3%. Our results also found negative effect size, although not significant, from 0 (Figure 5(a)). This is possibly caused by AMF-promoted photosynthesis mitigating the removal of carbon allocations.

For AMF-pathogen interactions, this study found reciprocal inhibition which depended on pathogen actioning modes and AMF taxonomic family (Figures 4 and 5). AMF had significantly harmful effects on both migratory and sedentary nematodes (Figure 4(b)). This is against one previous study of Borowicz [5], in which the author found that migratory nematodes benefited from mycorrhizal plants. Borowicz [5] admitted that this phenomenon might be caused by small sample size. Negative effects of AMF on fungal pathogens were dependent on actioning modes (Figure 4(c)). Root-rotting fungal pathogens had larger inhibition than shoot-wilt ones. The negative effects of AMF on nematodes and fungal pathogens could be explained by direct and indirect mechanisms. Direct mechanism was assumed that AMF compete with nematodes and fungal pathogens for infection sites or photosynthates and thus suppress their growth [24–26]. Indirect mechanisms were possibly mediated by several means. For example, AMF may induce defense responses, thus increasing host resistance [1, 29, 36]; altering root exudations utilized by nematodes and fungal pathogens [30]; or promoting other microbes which compete for resources with pathogens [31]. Our results of negative effects of pathogens on AMF suggested that the assumed direct mechanisms might play major roles. Irrespective of nematodes or fungal pathogens, Glomeraceae had smaller inhibition in their growth than Non-Glomeraceae. As we knows, Glomeraceae have higher and more rapid root colonization than other AMF families [34, 37]. Thus, Glomeraceae might have higher competitiveness than other AMF families when competing for carbon or infection sites with nematodes and fungal pathogens.

Above all, positive interactions between AMF and plants might be indirectly mediated by AMF suppressing biotic stressors of plants, or directly mediated by improving growth and function of roots to enhance plant tolerance [28]. These results also suggested high functional diversity of AMF [38]. Positive effects of AMF and negative effects of biotic stressors might form tradeoffs for plant growth. These multiple species interactions play potential roles in driving the dynamics of plant community responding to environmental changes and maintaining ecosystem stability [1]. However, all above we obtained are phenomena. In the future, we need to further investigate the mechanisms underlying them, such as AMF-induced chemical cross-talk among plants, herbivores, and soil biota.

## 5. Conclusions

This meta-analysis found reciprocal inhibition between AMF and nematodes as well as pathogenic fungi, but unidirectional inhibition of AMF on herbivores. AMF negative effects on biotic stressors depended on feeding sites of herbivores and actioning modes of fungal pathogens, but not on feeding modes of nematodes. Negative effects of pathogens on AMF depended on AMF taxonomic levels. Thus, under attacks by herbivores, nematodes, and fungal pathogens, AMF significantly enhanced plant growth performance. Our study enhances the understanding of how species interactions playing their roles in assembling plant communities and controlling ecosystem processes and properties.

## Supplementary Material

Appedix 1: includes details of all these 144 source papers from which all the data used was collected.
Appedix 2: is details of datasets used for this meta-analysis,including sample number (Sample No.), first author name (Pub_1st_author), published year (Pub_year), Plant performance, Biotic stressor performance,Mycorrhizal performance, Fungal species, Fungal family, Biotic type,Biotic stressor species and Feeding sites/modes.
