# Supplementary material for: Meta-Analysis of Interactions between Arbuscular Mycorrhizal Fungi and Biotic Stressors of Plants
Source: ScientificWorldJournal. 2014 Jan 16;2014:746506. doi: 10.1155/2014/746506 (PMC3914602; doi:10.1155/2014/746506)
Supplement: Supplementary file 1 — Appedix 1: includes details of all these 144 source papers from which all the data used was collected. Appedix 2: is details of datasets used for this meta-analysis,including sample number (Sample No.), first author name (Pub_1st_author), published year (Pub_year), Plant performance, Biotic stressor performance,Mycorrhizal performance, Fungal species, Fungal family, Biotic type,Biotic stressor species and Feeding sites/modes. [file 746506.f1.pdf]

1. Abdalla ME, Abdel-Fattah GM (2000) Influence of the endomycorrhizal fungus Glomus mosseae on the development of peanut pod rot disease in Egypt. Mycorrhiza 10: 29-35
2. Akhtar MS, Siddiqui ZA (2007) Biocontrol of a chickpea root-rot disease complex with Glomus intraradices, Pseudomonas putida and Paenibacillus polymyxa. Australasian Plant Pathology 36: 175-180
3. Akhtar MS, Siddiqui ZA (2008) Biocontrol of a root-rot disease complex of chickpea by *Glomus* intraradices, Rhizobium sp and Pseudomonas straita. Crop Protection 27: 410-417
4. Akhtar MS, Siddiqui ZA (2008) *Glomus* intraradices, Pseudomonas alcaligenes, and Bacillus pumilus: effective agents for the control of root-rot disease complex of chickpea (Cicer arietinum L.). Journal of General Plant Pathology 74: 53-60
5. Akkopru A, Demir S (2005) Biological control of Fusarium wilt in tomato caused by Fusarium oxysporum f. sp lycopersici by AMF Glomus intraradices and some rhizobacteria. Journal of Phytopathology 153: 544-550
6. Alraddad AM (1995) Interaction of *Glomus mosseae* and *Paecilomyces lilacinus* on *Meloidogyne javanica* of tomato. Mycorrhiza 5: 233-236
7. Anjos ÉCT, Cavalcante UMT, Gonçalves DMC, Pedrosa EMR, Santos VF, Maia LC (2010) Interactions between an arbuscular mycorrhizal fungus (Scutellospora heterogama) and the root-knot nematode (Meloidogyne incognita) on sweet passion fruit (Passiflora alata). Brazilian Archives of Biology and Technology 53: 801-809
8. Atilano RA, Menge JA, Vangundy SD (1981) Interaction between *Meloidogyne arenaria* and *Glomus fasciculatus* in grape. Journal of Nematology 13: 52-57
9. Attia M, Hamed HA, Turky AS (2004) Influence of root colonization with bacillus subtilis, Trichoderma harzianum and arbuscular mycorrhizae on promoting tomato seedling, yield and protection against fusarium crown and root rot. Bulletin of the National Research Centre (Cairo) 29: 347-360
10. Azcón R, Barea JM (1997) Mycorrhizal dependency of a representative plant species in Mediterranean shrublands (Lavandula spica L.) as a key factor to its use for revegetation strategies in desertification-threatened areas. Applied Soil Ecology 7: 83-92
11. Bagyaraj D, Manjunath A, Reddy DDR (1979) Interaction of vesicular arbuscular mycorrhiza with root knot nematodes in tomato. Plant and Soil 51: 397-403
12. Basu MJ, Santhaguru K (2009) Impact of Glomus fasciculatum and fluorescent Pseudomonas on growth performance of Vigna radiata (l.) wilczek challenged with phytopathogens. Journal of Plant Protection Research 49: 190-194
13. Behn O (2008) Influence of Pseudomonas fluorescens and arbuscular mycorrhiza on the growth, yield, quality and resistance of wheat infected with Gaeumannomyces graminis. Journal of Plant Diseases and Protection 115: 4-8
14. Bennett AE, Bever JD (2007) Mycorrhizal species differentially alter plant growth and response to herbivory. Ecology 88: 210-218
15. Bennett AE, Bever JD (2009) Trade-offs between arbuscular mycorrhizal fungal competitive ability and host growth promotion in Plantago lanceolata. Oecologia 160: 807-816
16. Berta G, Sampo S, Gamalero E, Massa N, Lemanceau P (2005) Suppression of Rhizoctonia root-rot of tomato by Glomus mossae BEG12 and Pseudomonas fluorescens A6RI is associated with their effect on the pathogen growth and on the root morphogenesis. European Journal of Plant Pathology 111: 279-288

17. Boby VU, Bagyaraj DJ (2003) Biological control of root-rot of Coleus forskohlii Briq. using microbial inoculants. World Journal of Microbiology & Biotechnology 19: 175-180
18. Borowicz VA (1997) A fungal root symbiont modifies plant resistance to an insect herbivore. Oecologia 112: 534-542
19. Calvet C, Pera J, Barea JM (1993) Growth-response of marigold (*Tagetes erecta* l) to inoculation with Glomus mosseae, Trichoderma aureoviride and Pythium-ultimum in a peat perlite mixture. Plant and Soil 148: 1-6
20. Calvet C, Pinochet J, Camprubi A, Fernandez C (1995) Increased tolerance to the root-lesion nematode Pratylenchus-vulnus in mycorrhizal micropropagated ba-29 quince rootstock. Mycorrhiza 5: 253-258
21. Camprubi A, Pinochet J, Calvet C, Estaun V (1993) Effects of the root-lesion nematode Pratylenchus vulnus and the vesicular-arbuscular mycorrhizal fungus Glomus mosseae on the growth of three plum rootstocks. Plant and Soil 153: 223-229
22. Carling D, Roncadori R, Hussey R (1989) Interactions of vesicular-arbuscular mycorrhizal fungi, root-knot nematode, and phosphorus fertilization on soybean. Plant Disease 73: 730-733
23. Carling D, Roncadori R, Hussey R (1995) Interactions of arbuscular mycorrhizae, Meloidogyne arenaria, and phosphorus fertilization on peanut. Mycorrhiza 6: 9-13
24. Castellanos-Morales V, Keiser C, Cardenas-Navarro R, Grausgruber H, Glauninger J, Garcia-Garrido JM, Steinkellner S, Sampedro I, Hage-Ahmed K, Illana A, Ocampo JA, Vierheilig H (2011) The bioprotective effect of AM root colonization against the soil-borne fungal pathogen Gaeumannomyces graminis var. tritici in barley depends on the barley variety. Soil Biology & Biochemistry 43: 831-834
25. Castillo P, Nico AI, Azcon-Aguilar C, Rincon CDR, Calvet C, Jimenez-Diaz RM (2006) Protection of olive planting stocks against parasitism of root-knot nematodes by arbuscular mycorrhizal fungi. Plant Pathology 55: 705-713
26. Chakraborty MR, Ghosh I, Ojha S, Dutta S, Chatterjee NC (2008) Synergic effects of VAM and polyphenoloxidases on increased productivity of brinjal and tomato in relation to resistance against Fusarial wilt. Acta Phytopathologica et Entomologica Hungarica 43: 37-44
27. Chandanie W, Kubota M, Hyakumachi M (2009) Interactions between the arbuscular mycorrhizal fungus Glomus mosseae and plant growth-promoting fungi and their significance for enhancing plant growth and suppressing damping-off of cucumber (Cucumis sativus L.). Applied Soil Ecology 41: 336-341
28. Chandanie WA, Kubota M, Hyakumachi M (2006) Interactions between plant growth promoting fungi and arbuscular mycorrhizal fungus Glomus mosseae and induction of systemic resistance to anthracnose disease in cucumber. Plant and Soil 286: 209-217
29. Cimen I, Pirinc V, Sagir A, Akpinar C, Guzel S (2009) Effects of solarization and vesicular arbuscular mycorrhizal fungus (VAM) on phytopthora blight (Phytophthora capsici leonian) and yield in pepper. African Journal of Biotechnology 8: 4884-4894
30. Cordier C, Gianinazzi S, GianinazziPearson V (1996) Colonisation patterns of root tissues by Phytophthora nicotianae var parasitica related to reduced disease in mycorrhizal tomato. Plant and Soil 185: 223-232
31. Currie AF, Murray PJ, Gange AC (2011) Is a specialist root-feeding insect affected by arbuscular mycorrhizal fungi? Applied Soil Ecology 47: 77-83

32. D'Amelio R, Berta G, Gamalero E, Massa N, Avidano L, Cantamessa S, D'Agostino G, Bosco D, Marzachi C (2011) Increased plant tolerance against chrysanthemum yellows phytoplasma ('Candidatus Phytoplasma asteris') following double inoculation with Glomus mosseae BEG12 and Pseudomonas putida S1Pf1Rif. Plant Pathology 60: 1014-1022
33. Dar GH, Zargar MY, Beigh GM (1997) Biocontrol of Fusarium root rot in the common bean (Phaseolus vulgaris L) by using symbiotic Glomus mosseae and Rhizobium leguminosarum. Microbial Ecology 34: 74-80
34. Davis R, Menge J (1980) Influence of Glomus fasciculatus and soil phosphorus on Phytophthora root rot of citrus. Phytopathology 70: 447-452
35. Davis R, Menge J (1981) Phytophthora parasitica inoculation and intensity of vesicular-arbuscular mycorrhizae in citrus. New Phytologist: 705-715
36. Davis R, Menge J, Erwin D (1979) Influence of Glomus fasciculatus and soil phosphorus on Verticillium wilt of cotton. Phytopathology 69: 453-456
37. Declerck S, Risede JM, Rufyikiri G, Delvaux B (2002) Effects of arbuscular mycorrhizal fungi on severity of root rot of bananas caused by Cylindrocladium spathiphylli. Plant Pathology 51: 109-115
38. del Carmen Jaizme-Vega M, Rodriguez-Romero AS, Barroso Nunez LA (2006) Effect of the combined inoculation of arbuscular mycorrhizal fungi and plant growth-promoting rhizobacteria on papaya (Carica papaya L.) infected with the root-knot nematode Meloidogyne incognita. Fruits 61: 151-162
39. Deliopoulos T, Minnis ST, Jones PW, Haydock PPJ (2010) Enhancement of the efficacy of a carbamate nematicide against the potato cyst nematode, Globodera pallida, through mycorrhization in commercial potato fields. Journal of Nematology 42: 22-32
40. Diedhiou PM, Hallmann J, Oerke EC, Dehne HW (2003) Effects of arbuscular mycorrhizal fungi and a non-pathogenic Fusarium oxysporum on Meloidogyne incognita infestation of tomato. Mycorrhiza 13: 199-204
41. Elsen A, Baimey H, Sweenen R, De Waele D (2003) Relative mycorrhizal dependency and mycorrhiza-nematode interaction in banana cultivars (Musa spp.) differing in nematode susceptibility. Plant and Soil 256: 303-313
42. Elsen A, Beeterens R, Swennen R, De Waele D (2003) Effects of an arbuscular mycorrhizal fungus and two plant-parasitic nematodes on Musa genotypes differing in root morphology. Biology and Fertility of Soils 38: 367-376
43. Elsen A, Gervacio D, Swennen R, De Waele D (2008) AMF-induced biocontrol against plant parasitic nematodes in Musa sp.: a systemic effect. Mycorrhiza 18: 251-256
44. Filion M, St-Arnaud M, Jabaji-Hare SH (2003) Quantification of Fusarium solani f. sp phaseoli in mycorrhizal bean plants and surrounding mycorrhizosphere soil using real-time polymerase chain reaction and direct isolations on selective media. Phytopathology 93: 229-235
45. Forge T, Muehlchen A, Hackenberg C, Neilsen G, Vrain T (2001) Effects of preplant inoculation of apple (Malus domestica Borkh.) with arbuscular mycorrhizal fungi on population growth of the root-lesion nematode, Pratylenchus penetrans. Plant and Soil 236: 185-196
46. Fritz M, Jakobsen I, Lyngkjaer MF, Thordal-Christensen H, Pons-Kuehnemann J (2006) Arbuscular mycorrhiza reduces susceptibility of tomato to Alternaria solani. Mycorrhiza 16:

413-419

47. Gallou A, Lucero Mosquera HP, Cranenbrouck S, Pablo Suarez J, Declerck S (2011) Mycorrhiza induced resistance in potato plantlets challenged by Phytophthora infestans. Physiological and Molecular Plant Pathology 76: 20-26
48. Gange A, Brown V, Sinclair G (1994) Reduction of black vine weevil larval growth by vesicular-arbuscular mycorrhizal infection. Entomologia Experimentalis et Applicata 70: 115-119
49. Gange AC (2001) Species-specific responses of a root- and shoot-feeding insect to arbuscular mycorrhizal colonization of its host plant. New Phytologist 150: 611-618
50. Gange AC, Bower E, Brown VK (1999) Positive effects of an arbuscular mycorrhizal fungus on aphid life history traits. Oecologia 120: 123-131
51. Gange AC, Brown VK, Aplin DM (2003) Multitrophic links between arbuscular mycorrhizal fungi and insect parasitoids. Ecology Letters 6: 1051-1055
52. Gange AC, Brown VK, Aplin DM (2005) Ecological specificity of arbuscular mycorrhizae: evidence from foliar-and seed-feeding insects. Ecology 86: 603-611
53. Garmendia I, Goicoechea N, Aguirreolea J (2004) Antioxidant metabolism in asymptomatic leaves of Verticillium-infected pepper associated with an arbuscular mycorrhizal fungus. Journal of Phytopathology 152: 593-599
54. Garmendia I, Goicoechea N, Aguirreolea J (2004) Effectiveness of three Glomus species in protecting pepper (Capsicum annuum L.) against verticillium wilt. Biological Control 31: 296-305
55. Gernns H, Alten H, Poehling HM (2001) Arbuscular mycorrhiza increased the activity of a biotrophic leaf pathogen-is a compensation possible? Mycorrhiza 11: 237-243
56. Goverde M, van der Heijden M, Wiemken A, Sanders I, Erhardt A (2000) Arbuscular mycorrhizal fungi influence life history traits of a lepidopteran herbivore. Oecologia 125: 362-369
57. Grandison GS, Cooper KM (1986) Interaction of vesicular-arbuscular mycorrhizae and cultivars of alfalfa susceptible and resistant to Meloidogyne hapla. Journal of nematology 18: 141
58. Hao ZP, Christie P, Qin L, Wang CX, Li XL (2005) Control of fusarium wilt of cucumber seedlings by inoculation with an arbuscular mycorrhical fungus. Journal of Plant Nutrition 28: 1961-1974
59. Heald CM, Bruton BD, Davis RM (1989) Influence of Glomus intraradices and soil-phosphorus on Meloidogyne incognita infecting Cucumis melo. Journal of Nematology 21: 69-73
60. Hempel S, Stein C, Unsicker SB, Renker C, Auge H, Weisser WW, Buscot F (2009) Specific bottom-up effects of arbuscular mycorrhizal fungi across a plant-herbivore-parasitoid system. Oecologia 160: 267-277
61. Hoffmann D, Vierheilig H, Riegler P, Schausberger P (2009) Arbuscular mycorrhizal symbiosis increases host plant acceptance and population growth rates of the two-spotted spider mite Tetranychus urticae. Oecologia 158: 663-671
62. Hu J-L, Lin X-G, Wang J-H, Shen W-S, Wu S, Peng S-P, Mao T-T (2010) Arbuscular Mycorrhizal Fungal Inoculation Enhances Suppression of Cucumber Fusarium Wilt in Greenhouse Soils. Pedosphere 20: 586-593

63. Hussey R, Roncadori R (1978) Interaction of Pratylenchus brachyurus and Gigaspora margarita on cotton. Journal of nematology 10: 16
64. Hwang SF, Chakravarty P, Prevost D (1993) Effects of rhizobia, metalaxyl, and VA mycorrhizal fungi on growth, nitrogen-fixation, and development of pythium root-rot of sainfoin. Plant Disease 77: 1093-1098
65. Hwang SF, Chang KF, Chakravarty P (1992) Effects of vesicular-arbuscular mycorrhizal fungi on the development of *Verticillium* and *Fusarium* wilts of alfalfa. Plant Disease 76: 239-243
66. Jaiti F, Meddich A, El Hadrami I (2007) Effectiveness of arbuscular mycorrhizal fungi in the protection of date palm (Phoenix dactylifera L.) against bayoud disease. Physiological and Molecular Plant Pathology 71: 166-173
67. Jaizme-Vega M, Tenoury P, Pinochet J, Jaumot M (1997) Interactions between the root-knot nematode Meloidogyne incognita and Glomus mosseae in banana. Plant and Soil 196: 27-35
68. Jaizme-Vega MC, Pinochet J (1997) Growth response of banana to three mycorrhizal fungi in Pratylenchus goodeyi infested soil. Nematropica 27: 69-76
69. Jalaluddin M, Hajra NB, Firoza K, Shahina F (2008) Effect of Glomus callosum, Meloidogyne incognita and soil moisture on growth and yield of sunflower. Pakistan Journal of Botany 40: 391-396
70. Kasiamdari RS, Smith SE, Smith FA, Scott ES (2002) Influence of the mycorrhizal fungus, Glomus coronatum, and soil phosphorus on infection and disease caused by binucleate Rhizoctonia and Rhizoctonia solani on mung bean (Vigna radiata). Plant and Soil 238: 235-244
71. Khaosaad T, Garcia-Garrido JM, Steinkellner S, Vierheilig H (2007) Take-all disease is systemically reduced in roots of mycorrhizal barley plants. Soil Biology & Biochemistry 39: 727-734
72. Khatun S, Chatterjee NC (2011) Glomus fasciculatum in defense responses to fusarial wilt of Coleus forskohlii. Acta Agriculturae Scandinavica Section B-Soil and Plant Science 61: 136-142
73. Kobra N, Jalil K, Youbert G (2011) Arbuscular mycorrhizal fungi and biological control of Verticillium-wilted cotton plants. Archives of Phytopathology and Plant Protection 44: 933-942
74. Krishna H, Das B, Attri BL, Grover M, Ahmed N (2010) Suppression of Botryosphaeria canker of apple by arbuscular mycorrhizal fungi. Crop Protection 29: 1049-1054
75. Larsen J, Ravnskov S, Jakobsen I (2003) Combined effect of an arbuscular mycorrhizal fungus and a biocontrol bacterium against Pythium ultimum in soil. Folia Geobotanica 38: 145-154
76. Larsen J, Yohalem D (2004) Interactions between mycorrhiza and powdery mildew of cucumber. Mycological Progress 3: 123-128
77. Lax P, Becerra AG, Soteras F, Cabello M, Doucet ME (2011) Effect of the arbuscular mycorrhizal fungus Glomus intraradices on the false root-knot nematode Nacobbus aberrans in tomato plants. Biology and Fertility of Soils 47: 591-597
78. Lingua G, D'Agostino G, Massa N, Antosiano M, Berta G (2002) Mycorrhiza-induced differential response to a yellows disease in tomato. Mycorrhiza 12: 191-198
79. Liu RJ (1995) Effect of vesicular-arbuscular mycorrhizal fungi on verticillium wilt of cotton. Mycorrhiza 5: 293-297

80. Liu RJ, Li HF, Shen CY, Chiu WF (1995) Detection of pathogenesis-related proteins in cotton plants. Physiological and Molecular Plant Pathology 47: 357-363

81. MacGuidwin A, Bird G, Safir G (1985) Influence of Glomus fasciculatum on Meloidogyne hapla infecting Allium cepa. Journal of nematology 17: 389

82. Martinez-Medina A, Pascual JA, Lloret E, Roldan A (2009) Interactions between arbuscular mycorrhizal fungi and Trichoderma harzianum and their effects on Fusarium wilt in melon plants grown in seedling nurseries. Journal of the Science of Food and Agriculture 89: 1843-1850

83. Martínez-Medina A, Roldán A, Pascual JA (2011) Interaction between arbuscular mycorrhizal fungi and *Trichoderma harzianum* under conventional and low input fertilization field condition in melon crops: Growth response and Fusarium wilt biocontrol. Applied Soil Ecology 47: 98-105

84. Neeraj, Singh K (2010) Cyamopsis tetragonoloba L Taub inoculated with arbuscular mycorrhiza and Pseudomonas fluorescens and treated with mustard oil cake overcome Macrophomina root-rot losses. Biology and Fertility of Soils 46: 237-245

85. Neeraj, Singh K (2011) Organic amendments to soil inoculated arbuscular mycorrhizal fungi and Pseudomonas fluorescens treatments reduce the development of root-rot disease and enhance the yield of Phaseolus vulgaris L. European Journal of Soil Biology 47: 288-295

86. Newsham K, Fitter A, Watkinson A (1995) Arbuscular mycorrhiza protect an annual grass from root pathogenic fungi in the field. Journal of Ecology: 991-1000

87. Nogales A, Aguirreolea J, Maria ES, Camprubi A, Calvet C (2009) Response of mycorrhizal grapevine to Armillaria mellea inoculation: disease development and polyamines. Plant and Soil 317: 177-187

88. Nogales A, Camprubi A, Estaun V, Marfa V, Calvet C (2010) In vitro interaction studies between Glomus intraradices and Armillaria mellea in vines. Spanish Journal of Agricultural Research 8: S62-S68

89. Norman JR, Atkinson D, Hooker JE (1996) Arbuscular mycorrhizal fungal-induced alteration to root architecture in strawberry and induced resistance to the root pathogen Phytophthora fragariae. Plant and Soil 185: 191-198

90. O'Bannon J, Inserra R, Nemec S, Vovlas N (1979) The influence of Glomus mosseae on Tylenchulus semipenetrans-infected and uninfected Citrus limon seedlings. Journal of nematology 11: 247

91. O'Bannon J, Nemec S (1979) The response of Citrus limon seedlings to a symbiont, Glomus etunicatus, and a pathogen, Radopholus similis. Journal of nematology 11: 270

92. Ozgonen H, Akgul DS, Erkilic A (2010) The effects of arbuscular mycorrhizal fungi on yield and stem rot caused by Sclerotium rolfsii Sacc. in peanut. African Journal of Agricultural Research 5: 128-132

93. Ozgonen H, Erkilic A (2007) Growth enhancement and Phytophthora blight (Phytophthora capsici Leonian) control by arbuscular mycorrhizal fungal inoculation in pepper. Crop Protection 26: 1682-1688

94. Pandey R (2005) Management of Meloidogyne incognita in Artemisia pallens with bio-organics. Phytoparasitica 33: 304-308

95. Pandey R, Gupta ML, Singh HB, Kumar S (1999) The influence of vesicular-arbuscular mycorrhizal fungi alone or in combination with Meloidogyne incognita on Hyoscyamus niger

L. Bioresource Technology 69: 275-278

96. Pandey R, Kalra A, Gupta ML (2009) Evaluation of bio-agents and pesticide on root-knot nematode development and oil yield of patchouli. Archives of Phytopathology and Plant Protection 42: 419-423

97. Petit E, Gubler WD (2006) Influence of Glomus intraradices on black foot disease caused by Cylindrocarpon macrodidymum on Vitis rupestris under controlled conditions. Plant Disease 90: 1481-1484

98. Pinochet J, Calvet C, Camprubi A, Fernandez C (1995) Growth and nutritional response of nemared peach rootstock infected with *Pratylenchus vulnus* and the mycorrhizal fungus *Glomus mosseae*. Fundamental and Applied Nematology 18: 205-210

99. Pinochet J, Calvet C, Camprubi A, Fernandez C (1995) Interaction between the root-lesion nematode Pratylenchus vulnus and the mycorrhizal association of Glomus intraradices and Santa Lucia 64 cherry rootstock. Plant and Soil 170: 323-329

100. Pinochet J, Camprubi A, Calvet C (1993) Effects of the root-lesion nematode Pratylenchus vulnus and the mycorrhizal fungus Glomus mosseae on the growth of emla-26 apple rootstock. Mycorrhiza 4: 79-83

101. Pinochet J, Camprubi A, Calvet C, Fernandez C, Kabana RR (1998) Inducing tolerance to the root-lesion nematode Pratylenchus vulnus by early mycorrhizal inoculation of micropropagated myrobalan 29 C plum rootstock. Journal of the American Society for Horticultural Science 1223: 342-347

102. Pinochet J, Fernández C, de Carmen Jaizme M, Tenoury P (1997) Micropropagated banana infected with Meloidogyne javanica responds to Glomus intraradices and phosphorus. HortScience 32: 101-103

103. Rabie GH (1998) Induction of fungal disease resistance in Vicia faba by dual inoculation with Rhizobium leguminosarum and vesicular-arbuscular mycorrhizal fungi. Mycopathologia 141: 159-166

104. Ren L, Lou Y, Sakamoto K, Inubushi K, Amemiya Y, Shen Q, Xu G (2010) Effects of Arbuscular Mycorrhizal Colonization on Microbial Community in Rhizosphere Soil and Fusarium Wilt Disease in Tomato. Communications in Soil Science and Plant Analysis 41: 1399-1410

105. Roncadori R, Hussey R (1977) Interaction of the endomycorrhizal fungus Gigaspora margarita and root-knot nematode on cotton. Phytopathology 67: 1507-1511

106. Sankaranarayanan C, Sundarababu R (2010) Influence of application methods of arbuscular mycorrhiza Glomus mosseae in the bio-management of root knot nematode, Meloidogyne incognita on black gram (Vigna mungo L.) Hepper. Journal of Biological Control 24: 51-57

107. Siddiqui ZA, Akhtar MS (2007) Effects of AM fungi and organic fertilizers on the reproduction of the nematode Meloidogyne incognita and on the growth and water loss of tomato. Biology and Fertility of Soils 43: 603-609

108. Siddiqui ZA, Akhtar MS (2007) Biocontrol of a chickpea root-rot disease complex with phosphate-solubilizing microorganisms. Journal of Plant Pathology 89: 67-77

109. Siddiqui ZA, Akhtar MS (2008) Effects of fertilizers, AM fungus and plant growth promoting rhizobacterium on the growth of tomato and on the reproduction of root-knot nematode Meloidogyne incognita. Journal of Plant Interactions 3: 263-271

110. Siddiqui ZA, Akhtar MS (2009) Effects of antagonistic fungi, plant growth-promoting

rhizobacteria, and arbuscular mycorrhizal fungi alone and in combination on the reproduction of Meloidogyne incognita and growth of tomato. Journal of General Plant Pathology 75: 144-153

111. Siddiqui ZA, Mahmood I (1995) Biological-control of Heterodera cajani and Fusarium udum by Bacillus subtilis, Bradyrhizobium japonicum and Glomus fasciculatum on pigeonpea. Fundamental and Applied Nematology 18: 559-566

112. Siddiqui ZA, Mahmood I (1995) Some observations on the management of the wilt disease complex of pigeonpea by treatment with a vesicular arbuscular fungus and biocontrol agents for nematodes. Bioresource technology 54: 227-230

113. Siddiqui ZA, Mahmood I (1998) Effect of a plant growth promoting bacterium, an AM fungus and soil types on the morphometrics and reproduction of Meloidogyne javanica on tomato. Applied Soil Ecology 8: 77-84

114. Siddiqui ZA, Mahmood T (2001) Effects of rhizobacteria and root symbionts on the reproduction of Meloidogyne javanica and growth of chickpea. Bioresource Technology 79: 41-45

115. Sikes BA, Cottenie K, Klironomos JN (2009) Plant and fungal identity determines pathogen protection of plant roots by arbuscular mycorrhizas. Journal of Ecology 97: 1274-1280

116. Singh R, Parameswaran TN, Rao EVSP, Puttanna K, Kalra A, Srinivas KVNS, Bagyaraj DJ, Divya S (2009) Effect of arbuscular mycorrhizal fungi and Pseudomonas fluorescens on root-rot and wilt, growth and yield of Coleus forskohlii. Biocontrol Science and Technology 19: 835-841

117. Smith G, Kaplan D (1988) Influence of mycorrhizal fungus, phosphorus, and burrowing nematode interactions on growth of rough lemon citrus seedlings. Journal of nematology 20: 539

118. Smith G, Roncadori R, Hussey R (1986) Interaction of endomycorrhizal fungi, superphosphate, and Meloidogyne incognita on cotton in microplot and field studies. Journal of nematology 18: 208

119. Song YY, Cao M, Xie LJ, Liang XT, Zeng RS, Su YJ, Huang JH, Wang RL, Luo SM (2011) Induction of DIMBOA accumulation and systemic defense responses as a mechanism of enhanced resistance of mycorrhizal corn (Zea mays L.) to sheath blight. Mycorrhiza 21: 721-731

120. Srivastava R, Khalid A, Singh US, Sharma AK (2010) Evaluation of arbuscular mycorrhizal fungus, fluorescent Pseudomonas and Trichoderma harzianum formulation against Fusarium oxysporum f. sp lycopersici for the management of tomato wilt. Biological Control 53: 24-31

121. Steinkellner S, Hage-Ahmed K, García-Garrido JM, Illana A, Ocampo JA, Vierheilig H (2011) A comparison of wild-type, old and modern tomato cultivars in the interaction with the arbuscular mycorrhizal fungus Glomus mosseae and the tomato pathogen Fusarium oxysporum f. sp. lycopersici. Mycorrhiza: 1-6

122. Strobel N, Hussey R, Roncadori R (1982) Interactions of Vesicular-Arbuscular Mycorrhizal Fungi, Meloidogyne incognita, and Soil Fertility on Peach. Phytopathology 72: 690-694

123. Subhashini DV, Padmaja K (2010) Interaction between arbuscular mycorrhizal fungi and Pythium aphanidermatum in tobacco seedbeds. Journal of Biological Control 24: 70-74

124. Sukhada M, Manjula R, Rawal RD (2011) Evaluation of arbuscular mycorrhiza and other biocontrol agents against Phytophthora parasitica var. nicotianae infecting papaya (Carica

papaya cv. Surya) and enumeration of pathogen population using immunotechniques. Biological Control 58: 22-29

125. Sundaresan P, Raja NU, Gunasekaran P (1993) Induction and accumulation of phytoalexins in cowpea roots infected with a mycorrhizal fungusGlomus fasciculatum and their resistance toFusarium wilt disease. Journal of biosciences 18: 291-301

126. Tabin T, Arunachalam A, Shrivastava K, Arunachalam K (2009) Effect of arbuscular mycorrhizal fungi on damping-off disease in Aquilaria agallocha Roxb. seedlings. Tropical Ecology 50: 243-248

127. Talavera M, Itou K, Mizukubo T (2001) Reduction of nematode damage by root colonization with arbuscular mycorrhiza (Glomus spp.) in tomato-Meloidogyne incognita (Tylenchida : Meloidognidae) and carrot-Pratylenchus penetrans (Tylenchida : Pratylenchidae) pathosystems. Applied Entomology and Zoology 36: 387-392

128. Talavera M, Itou K, Mizukubo T (2002) Combined application of Glomus sp and Pasteuria penetrans for reducing Meloidogyne incognita (Tylenchida : Meloidogynidae) populations and improving tomato growth. Applied Entomology and Zoology 37: 61-67

129. Teixeira dos Anjos EC, Tiburcio Cavalcante UM, Correia Goncalves DM, Regis Pedrosa EM, dos Santos VF, Maia LC (2010) Interactions between an Arbuscular Mycorrhizal Fungus (Scutellospora heterogama) and the Root-knot Nematode (Meloidogyne incognita) on Sweet Passion Fruit (Passiflora alata). Brazilian Archives of Biology and Technology 53: 801-809

130. Thygesen K, Larsen J, Bodker L (2004) Arbuscular mycorrhizal fungi reduce development of pea root-rot caused by Aphanomyces euteiches using oospores as pathogen inoculum. European Journal of Plant Pathology 110: 411-419

131. Toussaint JP, Kraml M, Nell M, Smith SE, Smith FA, Steinkellner S, Schmiderer C, Vierheilig H, Novak J (2008) Effect of Glomus mosseae on concentrations of rosmarinic and caffeic acids and essential oil compounds in basil inoculated with Fusarium oxysporum f.sp basilici. Plant Pathology 57: 1109-1116

132. Utkhede R (2006) Increased growth and yield of hydroponically grown greenhouse tomato plants inoculated with arbuscular mycorrhizal fungi and Fusarium oxysporum f. Sp radicis-lycopersici. Biocontrol 51: 393-400

133. Vaast P, Caswell-Chen EP, Zasoski RJ (1998) Influences of a root-lesion nematode, Pratylenchus coffeae, and two arbuscular mycorrhizal fungi, Acaulospora mellea and Glomus clarum on coffee (Coffea arabica L.). Biology and Fertility of Soils 26: 130-135

134. Vicari M, Hatcher P, Ayres P (2002) Combined effect of foliar and mycorrhizal endophytes on an insect herbivore. Ecology 83: 2452-2464

135. Vigo C, Norman JR, Hooker JE (2000) Biocontrol of the pathogen Phytophthora parasitica by arbuscular mycorrhizal fungi is a consequence of effects on infection loci. Plant Pathology 49: 509-514

136. Vos C, Geerinckx K, Mkandawire R, Panis B, De Waele D, Elsen A (2012) Arbuscular mycorrhizal fungi affect both penetration and further life stage development of root-knot nematodes in tomato. Mycorrhiza 22: 157-163

137. Waceke JW, Waudo SW, Sikora R (2001) Suppression of Meloidogyne hapla by arbuscular mycorrhiza fungi (AMF) on pyrethrum in Kenya. International Journal of Pest Management 47: 135-140

138. Waceke JW, Waudo SW, Sikora R (2002) Effect of inorganic phosphatic fertilizers on the

efficacy of an arbuscular mycorrhiza fungus against a root-knot nematode on pyrethrum. International Journal of Pest Management 48: 307-313

139. Wamberg C, Christensen S, Jakobsen I (2003) Interaction between foliar-feeding insects, mycorrhizal fungi, and rhizosphere protozoa on pea plants. Pedobiologia 47: 281-287

140. Wooley SC, Paine TD (2007) Can intra-specific genetic variation in arbuscular mycorrhizal fungi (Glomus etunicatum) affect a mesophyll‐feeding herbivore (Tupiocoris notatus Distant)? Ecological Entomology 32: 428-434

141. Zambolim L, Schenck N (1983) Reduction of the effects of pathogenic, root-infecting fungi on soybean by the mycorrhizal fungus, Glomus mosseae. Phytopathology 73: 1402-1405

142. Zhang L, Zhang J, Christie P, Li X (2008) Pre-inoculation with arbuscular mycorrhizal fungi suppresses root knot nematode (Meloidogyne incognita) on cucumber (Cucumis sativus). Biology and Fertility of Soils 45: 205-211

143. Zhang L, Zhang J, Christie P, Li X (2009) Effect of Inoculation with the Arbuscular Mycorrhizal Fungus Glomus Intraradices on the Root-Knot Nematode Meloidogyne Incognita in Cucumber. Journal of Plant Nutrition 32: 967-979

144. Ziedan E-SH, Elewa IS, Mostafa HM, Sahab AF (2011) Application of mycorrhizae for controlling root diseases of sesame. Journal of Plant Protection Research 51: 355-361
